# Rapid detection of *Pseudomonas aeruginosa* using a DNAzyme‐based sensor

**DOI:** 10.1002/fsn3.2367

**Published:** 2021-06-11

**Authors:** Mingcan Qin, Xiaoyi Ma, Shihui Fan, Hangjie Wu, Wanli Yan, Xiaopeng Tian, Jing Lu, Mingsheng Lyu, Shujun Wang

**Affiliations:** ^1^ Jiangsu Key Laboratory of Marine Bioresources and Environment/Jiangsu Key Laboratory of Marine Biotechnology Jiangsu Ocean University Lianyungang China; ^2^ Co‐Innovation Center of Jiangsu Marine Bio‐industry Technology Jiangsu Ocean University Lianyungang China; ^3^ Jiangsu Marine Resources Development Research Institute Lianyungang China

**Keywords:** DNAzyme, *Pseudomonas aeruginosa*, rapid detection

## Abstract

In the present study, a DNAzyme was screened in vitro through the use of a DNA library and crude extracellular mixture (CEM) of *Pseudomonas aeruginosa*. Following eight rounds of selection, a DNAzyme termed PAE‐1 was obtained, which displayed high rates of cleavage with strong specificity. A fluorescent biosensor was designed for the detection of *P. aeruginosa* in combination with the DNAzyme. A detection limit as low as 1.2 cfu/ml was observed. Using proteases and filtration, it was determined that the target was a protein with a molecular weight of 10 kDa–50 kDa. The DNAzyme was combined with a polystyrene board to construct a simple indicator plate sensor which produced a color that identified the target within 10 min. The results were reliable when tap water and food samples were tested. The present study provides a novel experimental strategy for the development of sensors based on a DNAzyme to rapidly detect *P. aeruginosa* in the field.

## Graphical abstract

We successfully screened the DNAzyme (PAE‐1) with high specificity and sensitivity to directly detect *P. aeruginosa* without secondary elution. Our research carried out a sensor for monitoring and preventing foods contamination.

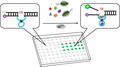

## 1 INTRODUCTION

*Pseudomonas aeruginosa* is a gram‐negative bacillus widely distributed in nature, and also a common conditional pathogen on human skin and in the respiratory tract. In 1882, *P. aeruginosa* was separated from the pus of a wound by Gersard for the first time and found able to survive in wet and dry environments (Woodford & Livermore, 2009). Tap water (Garvey et al., 2016; Gautam, 2021) and medical equipment (Alvarez‐Lerma et al., 2018) are often contaminated with *P. aeruginosa* which not only has innate resistance to a variety of antibiotics, it also has the propensity to develop drug resistance (Feng et al., 2019; Langendonk et al., 2021). *P. aeruginosa* accounts for 10%–15% of all nosocomial infections (Bassetti et al., 2018), especially those in intensive care unit (ICU) (Cohen et al., 2017; Coppry et al., 2020). Its pathogenic characteristics cause secondary infections, occurring mostly when the body's immunity is reduced, such as after extensive burns (Karaky et al., 2020) and following surgery (Richards & Marshall, 2018). *P. aeruginosa* can also cause human Hospital‐Acquired Pneumonia (Goncalves‐De‐Albuquerque et al., 2016), urinary tract infections (Pachori et al., 2019; Tumbarello et al., 2020), and bacteremia (Callejas‐Díaz et al., 2019; Mccarthy & Paterson, 2017). There are approximately 51,000 medical‐related infections in the United States every year (Cdc, 2019).

Many robust methods exist to detect bacteria, each having its own characteristics. Traditional culture methods (Rashno Taee et al., 2014) are highly accurate but are time‐consuming and require specific media and differentiation tests to identify them. Immunological assays (Huang et al., 2020; Lin, Zhou, & Tang, 2017) and polymerase chain reaction (PCR) (Guo et al., 2020) technology based on nucleic acid amplification greatly shortens the duration of identification. However, immunological assays (Krithiga et al., 2016; Shahdordizadeh et al., 2017) suffer from being expensive and complicated sample preparation steps. PCR technology (Aghamollaei et al., 2015) requires DNA extraction, and dead extracted bacteria can also produce false‐positive signals. Electrochemical methods (Lin, Zhou, Tang, Niessner, et al., 2017; Zhou & Tang, 2020) are highly sensitive but the external environment can easily cause interference (Sismaet et al., 2014) and additionally, requires specialized equipment. Methods allowing rapid detection are particularly useful.

Used widely as molecular tools, enzyme‐based methods can also be used in biosensing (Zhang et al., 2019), environmental detection (Wang et al., 2018), and medical diagnosis (Kumar et al., 2019; Zhou et al., 2017). DNAzyme is a functional nucleic acid (Peng et al., 2020), a single‐stranded DNA molecule with catalytic activity that can be isolated from random synthetic libraries by in vitro selection technology (Gu et al., 2019). It was reported that the first DNA was used for RNA cleavage (Breaker & Joyce, 1994) in 1994. Santoro and others obtained a classic Pb^2+^‐dependent 8–17 DNAzyme through in vitro screening (Santoro Sw, 1997). Subsequently, increasing numbers of metal ion‐dependent DNAzymes have been discovered (Li et al., 2017; Torabi et al., 2015; Yu et al., 2018). From the unique mixture (crude extracellular mixture, CEM) resulting from microbial reproductive metabolism, Yingfu Li developed the fluorescent DNAzyme probe RDF‐EC1 for the detection of *Escherichia coli* (Ali et al., 2011). M. Monsur Ali present a DNAzyme‐based fluorescent paper sensor for Klebsiella pneumoniae (Ali, Slepenkin, et al., 2019). A colorimetric paper DNAzyme sensor for *E. coli* and *Helicobacter pylori* has undergone continuous development (Ali, Wolfe, et al., 2019; Tram et al., 2014), providing rapid bacterial detection on site. Previous reports demonstrate the unique advantages of DNAzymes in bacterial detection (Mcconnell et al., 2020; Zhou et al., 2020).

*Pseudomonas aeruginosa* not only contaminates medical equipment, it also is an important pathogen in sources of water and food, posing a serious threat to human health. In this study, we focused on simple and efficient detection method of *P. aeruginosa*. By integrating previously reported selecting methods (Gu et al., 2019; Li, 2011; Zhang et al., 2016) with the unmodified DNA library and the crude extracellular mixture (CEM) of *P. aeruginosa*, a highly specific DNAzyme was obteined through conventional DNAzyme selection. The DNAzyme was attached to a polystyrene board to fabricate a DNAzyme sensor "mixed read" assay (Zhang et al., 2016) that could rapidly identify *P. aeruginosa*. This study describes an effective method for screening and making other bacterial DNAzyme sensors, which provides a basis for the establishment of related detection methods for *P. aeruginosa*.

## 2 RESULTS AND DISCUSSION

### 2.1 DNAzyme screening and sequence analysis

**FIGURE 1:**
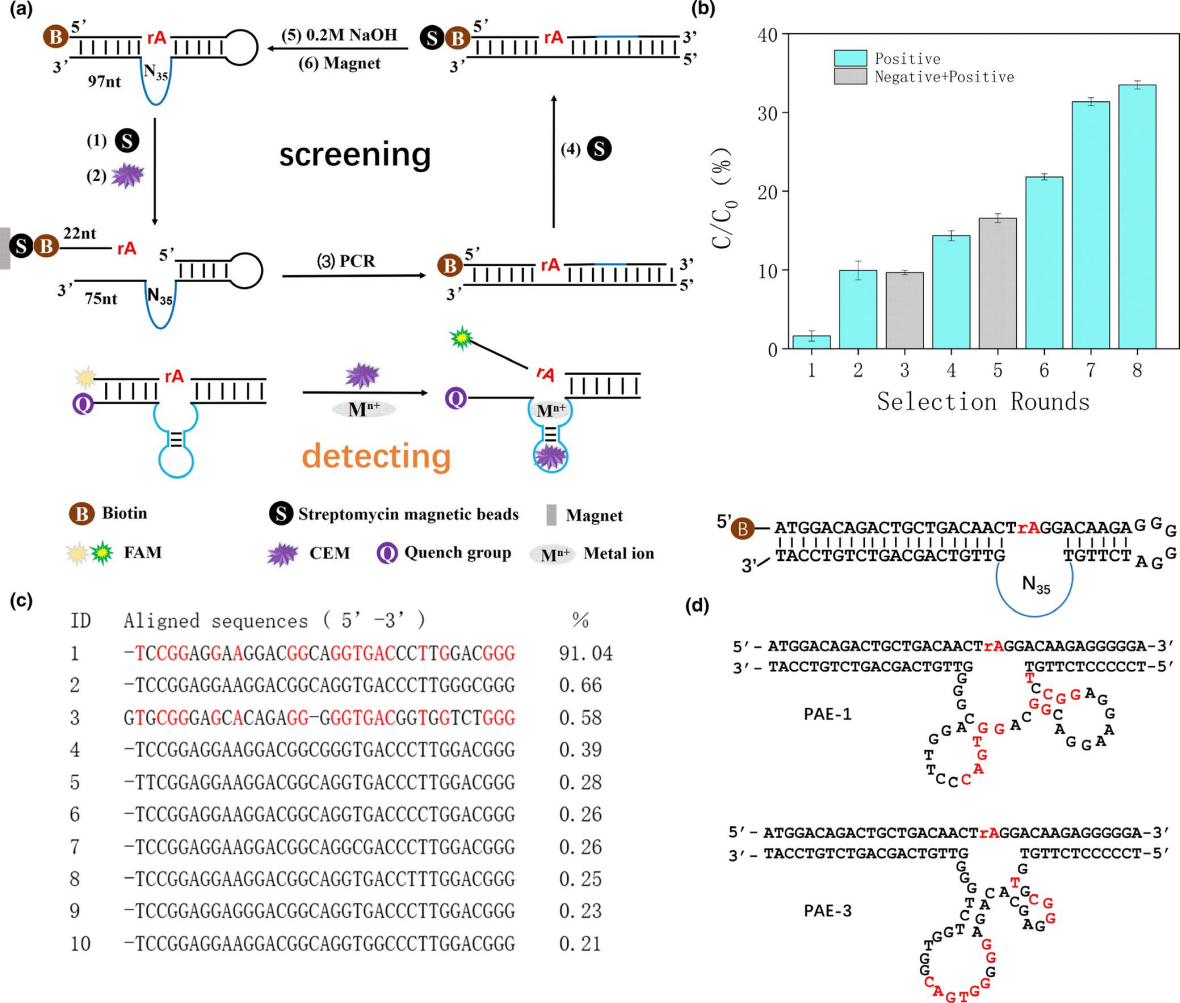
(a) CEM‐PA specific DNA enzyme selection process. The original library was fixed, the sequence was cleaved in the presence of CEM. After PCR, cleaved fragments were restored to the original full‐length library to inoculate the subsequent round of selection. A molecular beacon design was used for the DNAzyme sensor. (b) Ratio of cleavage of selection progress (C/C0). (c) The most abundant 10 DNA sequences using deep sequencing. Red letters in bold represent highly aligned PAE‐1 and PAE‐3 sequences. (d) The initial library selected in vitro. The cleavage site is at the rA junction and labeled with biotin at the 5′ end. Secondary structure of PAE‐1 and PAE‐3 DNAzymes

DNAzyme is a DNA‐based catalyst. It has the advantages of high catalytic efficiency, it is simple to synthesize and modify, and elicits no immune reaction. DNAzymes also display an interesting tertiary structure more stable than proteins. In the present study, *P. aeruginosa* extracellular product (CEM‐PA) was used as a screening target. Eight rounds of screening were performed (Figure 1a) using an initial library of 10^13^ ~ 10^15^ molecules containing 35 random bases and an adenine ribonucleoside (rA) cleavage site (Figure 1a). *Pseudomonas aeruginosa* CEM‐PA was used directly as a target, by passing target isolation and identification in terms of time saved and cost. Through negative selection, the DNA sequences displaying nonspecific or weak binding were removed, while positive selection enriched the target‐specific sequences (Figure 1b). Over the final three rounds, cleavage time was gradually reduced. This allowed DNAzyme to be screened‐out which could perform cleavage quickly. In addition to the first introduction of negative selection in the third round, the yield of cleavage in other rounds increased. After round 8, cleavage yield was close to 35%. The PCR products of the 8th round were purified and sequenced.

**FIGURE 2:**
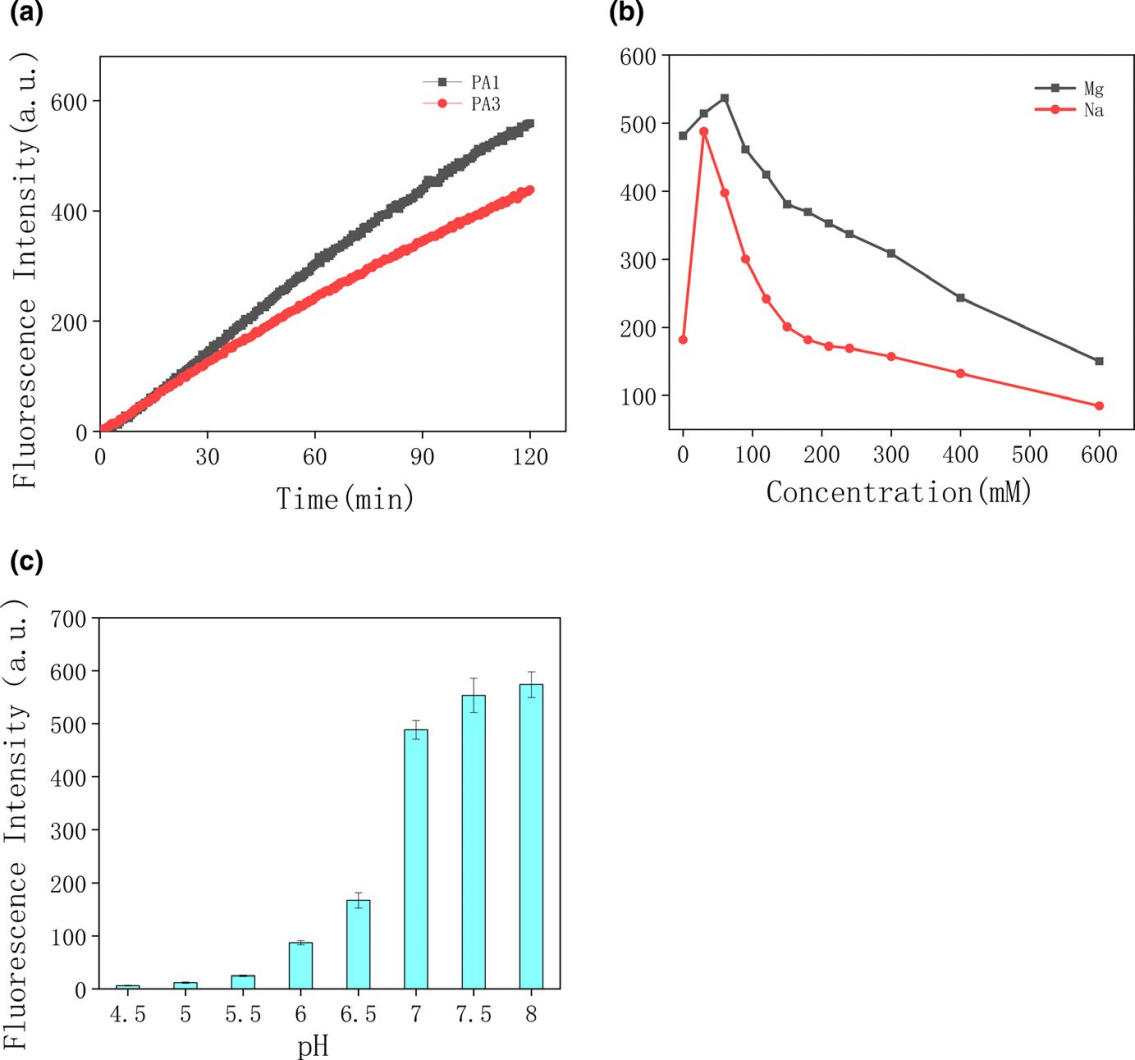
(a) Kinetics of PAE‐1 and PAE‐3. (b) Effects of Na^+^ and Mg^2+^ concentration on cleavage activity. (c) Cleavage at different pH values

Sangon Biotech (Shanghai, China) used the Miseq platform from Illumina for sequencing which generated 33,846 raw DNA reads. The first 10 sequences were selected by length and percentage, and compared with each other using Clustal software (Figure 1c). Except for the third sequence, only individual bases differed from each other in the other 9 sequences. Using IDT Oligo Analyzer 3.1 to predict the secondary structures of 1 and 3, termed PAE‐1 and PAE‐3, we found that both DNAzymes contained a special stem‐loop structure (Figure 1d). To determine their activity, the sequences of PAE‐1 and PAE‐3 were synthesized. Using dynamic analysis, PAE‐1 had greater cleavage activity (Figure 2a). The sequence of the red section of the stem ring was highly conserved on PAE‐1 and PAE‐3, possibly related to the active region of DNAzyme. From the predicted secondary structure, the stem rings were arranged more intensively on PAE‐1, possibly the reason for its high cleavage activity.

### 2.2 Influence of metal ions and pH on DNAzyme cleavage

The results demonstrate that the DNAzyme displayed the greatest cleavage activity at a Na^+^ concentration of 30 mM and Mg^2+^ concentration of 60 mM (Figure 2b). With increasing metal ion concentration, DNAzyme activity decreased sharply. This was possibly because the high concentration changed the three‐dimensional structure of the DNAzyme and reduced cleavage activity.

Previous studies have shown that pH also affects DNAzyme activity (Ma et al., 2019; Ma & Liu, 2019). As displayed in Figure 2c, the lytic activity of the DNAzyme gradually increased with increasing pH. Too high a pH should not be used because Mg^2+^ can easily precipitate in alkaline conditions, affecting the accuracy of the experiment (Figure 2c). Therefore, for subsequent experiments, a pH of 8.0 was selected.

### 2.3 CEM composition

**FIGURE 3:**
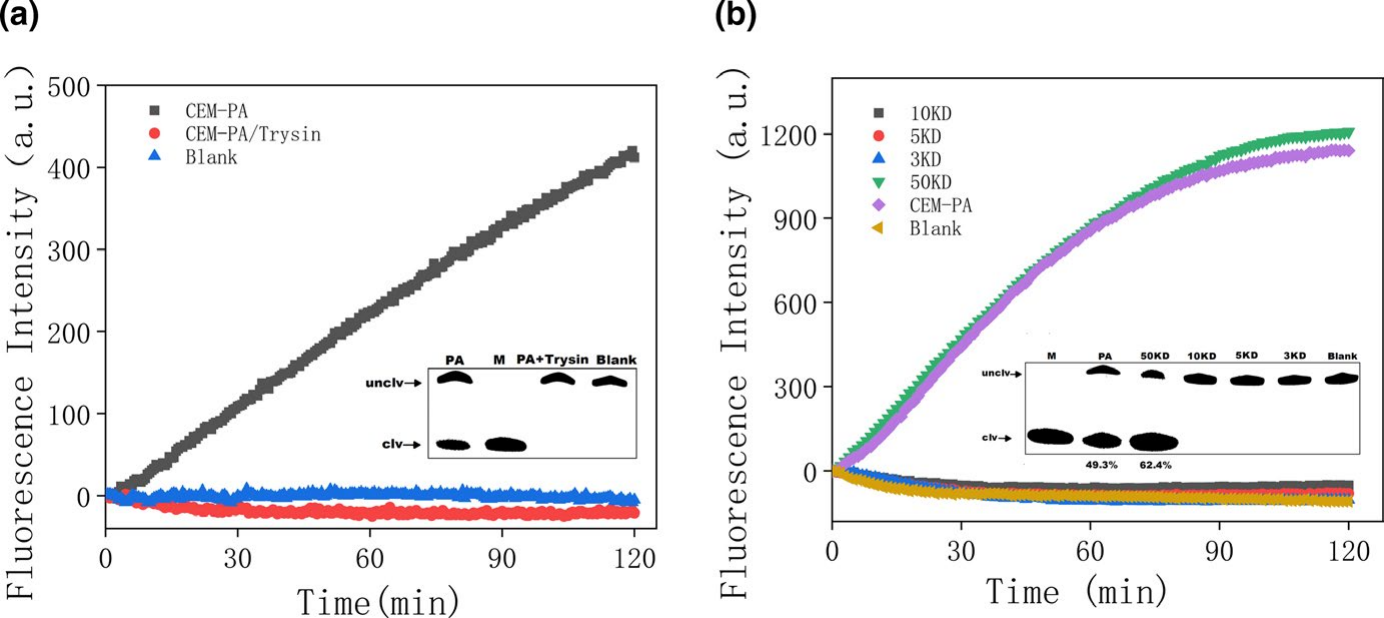
(a) Trypsin‐treated CEM‐PA demonstrated no signal from the PAE‐1 based biosensor. Inset: gel micrograph displaying activity. M: DNAzyme was completely cleaved by *P. aeruginosa* (b) Assessment of the molecular weight of the target protein. Inset: gel images for each part

Crude extracellular mixture is a complex extracellular mixture, in which it is difficult to identify an effective target molecule. Li and co‐workers discovered that DNAzyme cleavage was related to a transcription factor (protein) (Shen et al., 2016). It was first assumed that the target was a protein. CEM‐PA was treated with trypsin to digest the proteins, or boiled in trypsin for 10 min to inactivate it, then mixed them with PAE‐1 DNAzyme. Gel images and fluorescence‐based sensors indicated that Trypsin‐treated CEM‐PA demonstrated no signal from the PAE‐1 (Figure 3a). Therefore, the target may be a protein. An estimate of the molecular weight of the protein was performed by filtering the target through multiple spin columns (3 kDa–50 kDa). The results indicate that only the 50 kDa filtrate was able to induce cleavage of PAE‐1 DNAzyme (Figure 3b). Therefore, the molecular weight of the target protein was between 10 kDa and 50 kDa.

### 2.4 Highly specific detection

Quenchants were labeled at the 3′ end of the PAE‐1 DNAzyme chain, and a FAM fluorescent group was labeled at the 5′ end of the substrate (Du, Li, Yang, et al., 2020; Wang et al., 2020). When annealed to form the DNAzyme, the fluorescence was quenched (Figure 1a). The DNAzyme was reacted to cleaved, and the fluorescence signal was released when the CEM‐PA was added. We fixed the DNAzyme was fixed on polystyrene boards by dropping and drying naturally. Fluorescence could be observed by naked eye under blue light (490 nm).

**FIGURE 4:**
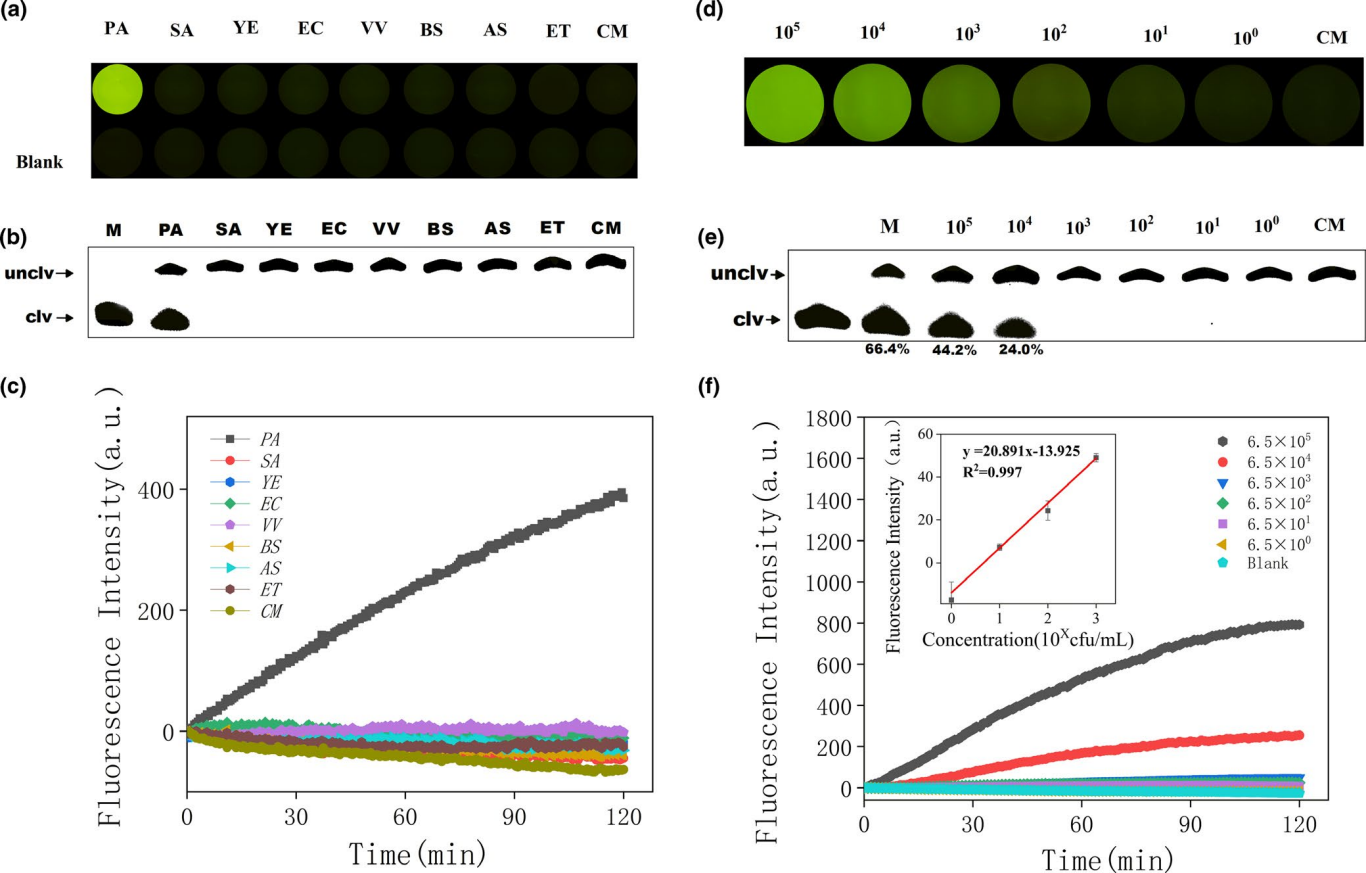
(a) Iindicator plate images of PAE‐1 specific detection after 10 min. PA: *Pseudomonas aeruginosa*; SA: *Staphylococcus aureus*; YE: *Yersinia enterocolitica*; EC: *Escherichia coli*; VV: *Vibrio vulnificus*; BS: *Bacillus subtilis*; AS: *Aeromonas salmonicida*; ET: *Edwardsiella tarda*; CM: Blank complete medium. (b) Image of cleavage gel for PAE‐1 specificity detection within 2 hr. (c) Biosensor‐based assay of PAE‐1 in blank culture medium and seven different bacteria. Gel image (e), indicator plate (d), and biosensor measurement (f) of PAE‐1 in different concentrations of *Pseudomonas aeruginosa* in CEM‐PA. (f) Fluorescence values of *P. aeruginosa* at different concentrations (Blank: ddwater without *P. aeruginosa*). Inset: Analytical calibration curve of fluorescence values of *P. aeruginosa* (concentrations of 6.5, 6.5 × 10, 6.5 × 10^2^, 6.5 × 10^3^) for 2 hr

In the present study, CEM‐PA was selected as a screening target. In addition to cell metabolism, the culture medium was also a key factor affecting the results. To eliminate the possible cleavage occurring due to the medium, blank medium was incubated with the PAE‐1 DNAzyme. In addition, cleavage conditions for PAE‐1 DNAzyme in 7 different bacterial extracellular products were also verified. As shown in Figure 4c, the results indicate that the blank medium (labeled blank control) not only failed to allow cleavage but also resulted in quenching of fluorescence. None of the seven bacteria displayed cleavage activity when tested. Only *V. vulnificus* fluorescence remained unchanged, while the fluorescence values of other bacteria decreased. It is possible that particular ions within the beef paste in the culture medium or CEM produced by these bacteria caused fluorescence quenching. The same results were obtained denatured polyacrylamide gel electrophoresis (Figure 4b). The DNAzyme can form loops which can bind with targets specifically through hydrophobic, electrostatic, hydrogen bonding, van der Waals force, base stacking, and shape complementarity (Gu et al., 2019; Zhang et al., 2016). The binding mechanism is similar with antibodies or aptamers which bind with targets. The combination of antibody and antigen is mainly determined by the complementarity of spatial structure. For the DNAzyme, generally, one ring of DNAzyme binds to metal ions, and another ring specifically binds to the targets. The DNAzyme only can be cleaved when both the metals and targets are existed. Electrostatic, hydrogen bonding, and hydrophobic interaction promote their combination between DNAzyme and targets (Jin et al., 2021; Park et al., 2021). Aptamers are also called "artificial antibodies" (Agyei et al., 2018), aptamers and DNAzymes can form a tertiary structure and specifically binds to the target (Cui et al., 2020; Kalra et al., 2018).

### 2.5 Highly sensitive detection

To evaluate the effective detection range of PAE‐1 DNAzyme sensor, 10‐fold serial dilutions of *P. aeruginosa* bacterial suspensions were produced. The number of *P. aeruginosa* in the present study was determined using a gradient dilution plate method from 6.5 × 10^8^ cfu/ml. When 30 μl CEM‐PA was added to 50 μl of detection system on the indicator board sensor, the fluorescence was increased with the concentration of *P. aeruginosa* (Figure 4d). Using PAE‐1 DNAzyme sensor, 10 μL CEM‐PA was added to 100 μl of detection system, resulting in a detection limit calculated to be 1.2 cfu/ml (Figure 4f insert). Using dPAGE, 10 μl CEM‐PA added to 50 μl of detection system resulted a clear band at 1.3 × 10^4^ cfu/ml (Figure 3e). We conclude that the fluorescence sensor has the lowest detection limit when using PAE‐1 DNAzyme sensor.

### 2.6 Cleavage ratio

**FIGURE 5:**
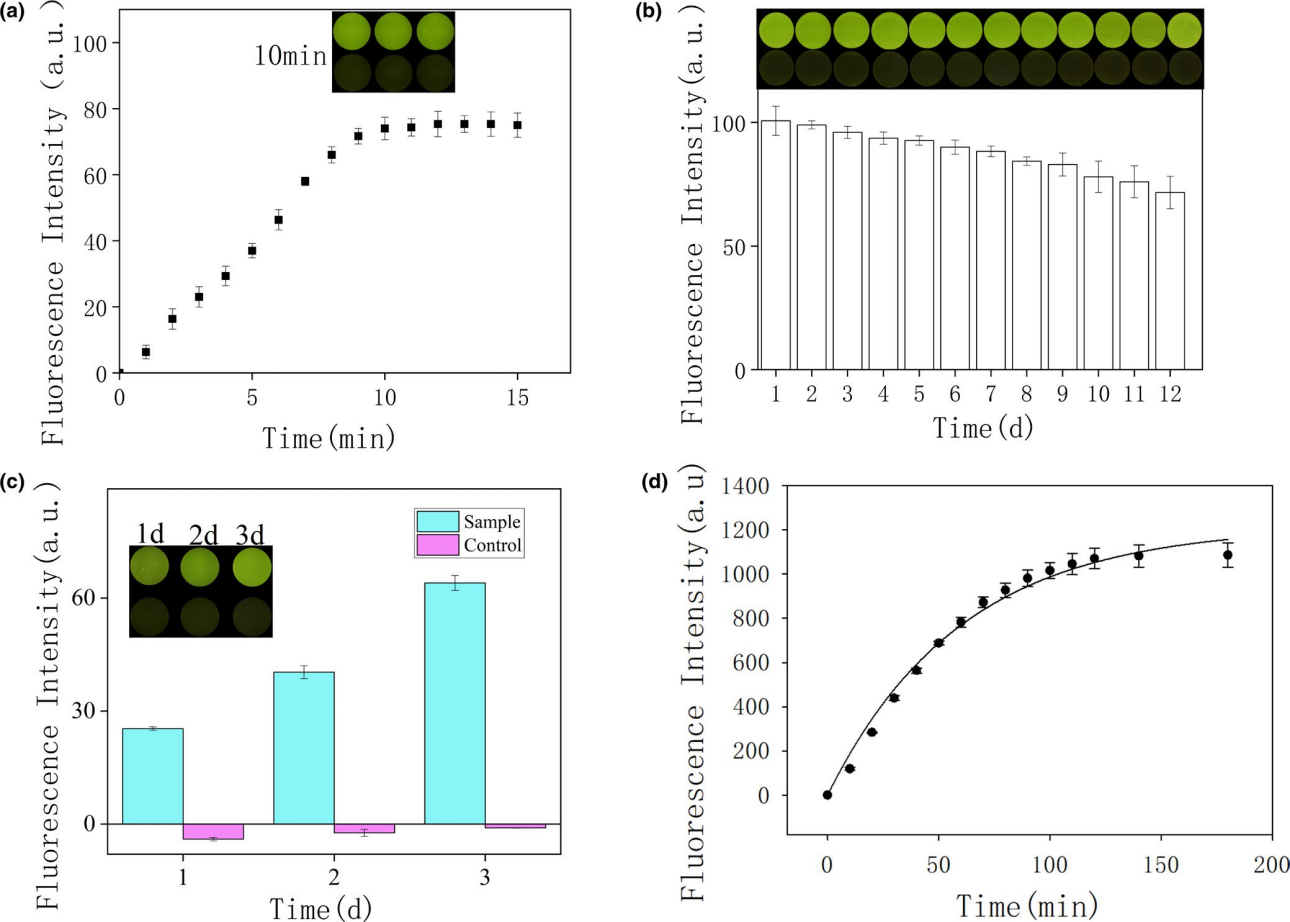
(a) Kinetics of the indicator plate sensor over 15 min. Inset: image of the indicator board sensor after 10 min. (b) Fluorescence intensity of the indicator board sensor after 12 days. Inset: corresponding indicator board sensor image. (c) Comparison of DNAzyme cleavage activity between control and sample. Inset: Image of the indicator board sensor after 10 min after 3 days. (d) The fluorescence intensity of PAE‐1 DNAzym in 3 hr. The result of the fitted curve indicates the cleavage rate of PAE‐1 DNAzyme

The first‐order reaction rate equation *Y_t_* = *Y*_0_ + a (1−e^−bx^) was used to conduct dynamic linear curve fitting, in which *Y_t_* and *Y*_0_ represented the fragment intensity of DNAzyme cleavage for reaction times of *t* and 0, respectively, where B represented the reaction rate constant. Results of fitting indicated that PAE‐1 DNAzyme displayed good cleavage performance, with a reaction rate constant of 0.0167 min^−1^ (Figure 5d). This rate indicates that the DNAzyme was effective in identifying *P. aeruginosa* using particular conditions.

### 2.7 Indicator board sensor

We optimized the detection time of PA‐1 DNAzyme on the indicator board sensor. According to the fluorescence value detected by the full‐wavelength microplate reader (Figure 5a), with the extension of time, the fluorescence value of PA‐1 DNAzyme keeps increasing, and the fluorescence value remains basically unchanged after 10 min. This may indicate that the extracellular product CEM‐PA of *P. aeruginosa* has completely cleaved DNAzyme. The dried DNAzyme on the polypropylene vinyl board could quickly reacted when the CEM‐PA was added. After 10 min, the DNAzyme was completely cleaved by CEM‐PA, and the fluorescence value reached maximum. Meanwhile, the controls could be distinguished by naked eyes under blue light. It indicated that the DNAzyme could maintain it basic structure and recover its reaction from dry state accompanied with CEM‐PA solution. So, the drying did not affect the function of DNAzyme. We also tested the storage time of the PA‐1 DNAzyme‐based biosensor. According to the double verification of the full‐wavelength microplate reader and the biosensor (Figure 5b), with the passage of time, the fluorescence value of PA‐1 DNAzyme only slightly decreased, which basically remained unchanged, indicating that the DNAzyme‐based biosensor can be stored for at least 12 days.

Tap water was added to the *P. aeruginosa* as the test group and tap water as the control group and tested over 3 days. As time progressed, the signal of the sensor in the experimental group increased gradually, while that of the control group remained essentially unresponsive (Figure 5c). The experiment indicated that the indicator board sensor quickly detected real samples. The present study demonstrates a device able to rapidly determine the presence of *P. aeruginosa* in remote areas. *P. aeruginosa* not only contaminates medical equipment, but also is widely present in tap water. *P. aeruginosa* poses a serious threat to human health, and its identification has been the subject of various studies (Kaur et al., 2015; Zhong et al., 2018). In the present study, a novel strategy was developed for rapid and convenient detection of *P. aeruginosa*.

### 2.8 Detection of *P. aeruginosa* in food

**FIGURE 6:**
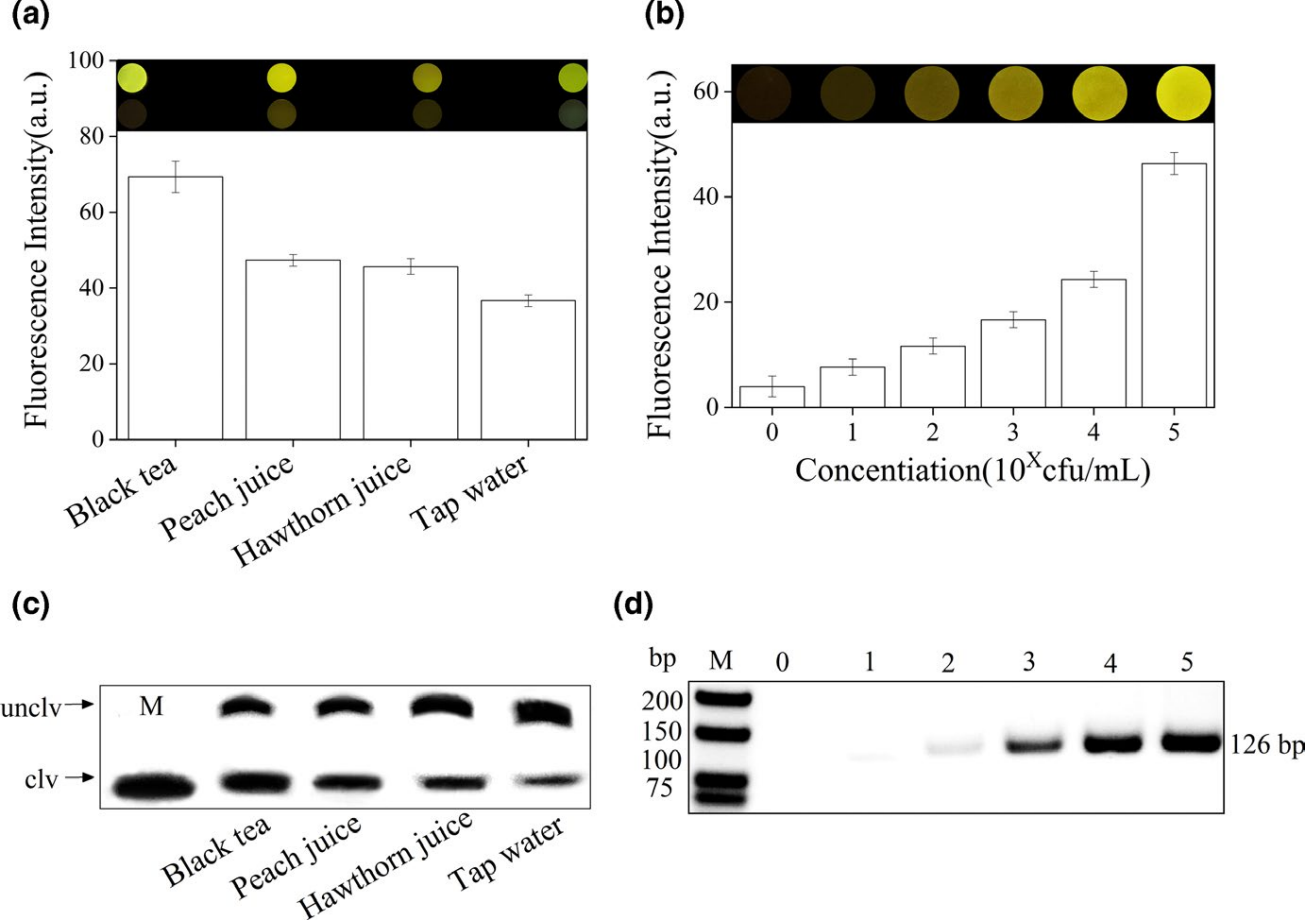
(a) Black tea, peach juice, hawthorn juice, and tap water artificially contaminated with *P. aeruginosa*, with uncontaminated samples as controls. Using a blue‐light transilluminator, fluorescence values are displayed equal to the experimental group minus the control group. (b) Fluorescence value and brightness of 6.9 cfu/ml–6.9 × 10^5^ cfu/ml *P. aeruginosa* in black tea. (c) Detection of *P. aeruginosa* in denaturing polyacrylamide gel. M: DNAzyme was completely cleaved by *P. aeruginosa*. (d) PCR amplification results of 69 cfu/ml–6.9 × 10^5^ cfu/ml *P. aeruginosa*

Black tea contaminated with *P. aeruginosa* contrasted greatly with uncontaminated samples and was easily detected using a blue‐light transilluminator (Figure 6a). Although the brightness of the contaminated peach juice was similar to that of black tea, the background light of the control group was also very bright, so it could not be observed directly. From the brightness of the indicator board sensor, the detection limit of *P. aeruginosa* in black tea was 6.9 × 10^2^ cfu/ml (Figure 6b). The results of gel electrophoresis showed the DNAzyme could be cleaved when the *P. aeruginosa* was existed in the samples of black tea, peach juice, hawthorn, and tap water (Figure 6c). The fragment of DNAzyme could be observed in the polyacrylamide gel. DNA for PCR was extracted from 69 cfu/ml ‐ 6.9 × 10^5^ cfu/ml *P. aeruginosa* in black tea, and 2% agarose gels showed that *P. aeruginosa* DNA remained close to 126 bp (Figure 6d). The results of both were consistent. The DNAzyme‐based sensor was able to detect 6.9 × 10^2^ cfu/ml *P. aeruginosa* with in 10 min.

## 3 CONCLUSIONS

Here, we obtained the DNAzyme by in vitro selection that was effective for the detection of *P. aeruginosa*. Compared with the previous strategy, the DNAzyme sensor had higher sensitivity. The detection of limit could reach 1.2 cfu/ml. In the pekoe tea contaminated by *P. aeruginosa*, the detection of limit was 6.9 × 10^2^ cfu/ml. In particularly, the methods did not require equipment and sample pretreatment. The detection of *P. aeruginosa* could be performed in site and obtained the results within 10 min. The DNAzyme sensors also could be easily applied by nontechnicians. For the limitations of DNAzyme sensors, they may interrupt by high concentration metals or extreme acidity and alkalinity in matrix. DNAzymes rely on specific auxiliary metal ions. The background of fluorescence could be affected by the matrix of samples (Cepeda‐Plaza & Peracchi, 2020; Cozma et al., 2021). And now, DNAzymes sensors applied in vitro only (Lake et al., 2019). The results provide the possibility of using the DNAzyme to reduce health risks and develop fast and inexpensive biosensors.

## 4 MATERIALS AND METHODS

### 4.1 Materials

*Pseudomonas aeruginosa, Yersinia enterocolitica, Aeromonas salmonicida, Vibrio anguillarum, V. vulnificus*, and *Edwards tarda* were bought from the China Center of Industrial Culture Collection (CICC). *Staphylococcus aureus, E. coli*, and *Bacillus subtilis* were from the Marine Resources Development Institute of Jiangsu (Lianyungang). Sequences of all oligonucleotides (Table 1) were synthesized by Sangon Biotech (Shanghai, China). Beef extract, Tween‐20, Ethanol absolute, Boracic acid, Tris(hydroxymethyl)aminomethane, NaOH, NaCl, MgCl_2_, KCl, and the metal salts were purchased from Sinopharm (China). N‐(2‐Hydroxyethyl)piperazine‐N′‐(2‐ ethanesulfonic acid) (HEPES) and 2‐(N‐morpholino) ethanesulfonic acid (MES) were purchased from Aladdin (China). Deoxynucleotide (dNTP) mix, Taq DNA polymerase with 10×PCR buffer, 6×gel loading dye, and low molecular weight DNA ladder were obtained from New England Biolabs (Shanghai, China). Agarose was acquired from Biowest (Chai wan, Hong Kong). Tryptone and yeast extract powder were obtained from Oxoid (Hampshire, England). Buffer I was 10 mM Tris‐HCL (pH7.5), 1 mM EDTA, 1 M NaCL, 0.01% ~ 0.1% Tween‐20, Buffer II was 100 Mm HEPES (pH7.5), 300 mM NaCL, 30 mM MgCL_2_, 0.02% Tween‐20. Distilled deionized water (ddwater 18.5 MΩ cm, MilliQ system) was used. All reagents were of analytical grade.

**TABLE 1:** DNA Sequences for in vitro Selection^a^

DNA name	DNA sequence (5′−3′)
Lib‐N35‐pool	pGGACAAGAGGGGGATCTTGT‐N_35_‐GTTGTCAGCAGTCTGTCCAT
Primer 1 (P1)	ATGGACAGACTGCTGACAAC
Primer 2 (P2)	Biotin‐TGCTGACAACTrAGGACAAGAGGGGGA
FAM‐substrate	FAM‐ATGGACAGACTGCTGACAACTrAGGACAAGAGGGGGA

Note

DNAzyme is a functional nucleic acid, a single‐stranded DNA molecule with catalytic activity, that can be isolated from random DNA libraries by selection technology. We successfully screened the DNAzyme (PAE‐1) with high specificity and sensitivity to directly detect *P. aeruginosa* without secondary elution. The PAE‐1 could detect *P. aeruginosa* at 1.2 cfu/ml in 10 min using our designed DNAzyme‐based sensor.

^a^ p: phosphorylation; N35: 35 random nucleotides; The ribose adenine cleavage site is represented by rA; FAM: Carboxyfluorescein.

### 4.2 Methods

#### 4.2.1 Preparation of screening library

The random library and its corresponding PCR primers were designed. Primer 1 was designed first, and then, primer 2 was designed according to primer 1. The 5 ′end of primer 2 contains biotin and a cleavage site in the middle. The library was constructed by primer 1 and primer 2. In the middle of the library are 35 random sequences randomly generated by Sangon Biotech (Shanghai, China). The 5 ′end of the library is phosphorylated. Primer Premier 5.0 software was used to design primer sequences to avoid hairpin formations and dimers, maximizing PCR efficiency. DNA sequences used in the experiments are listed in Table 1.

The initial library was prepared by PCR for screening for the DNAzyme. PCR reaction mixtures (50 μl) included cleaved DNA product (starting with Lib‐N35‐pool, 2 ~ 100 ng/μl), 1 μl each of forward primer (10 μmol/L) and reverse primer (10 μmol/L), 3 μl MgCl_2_ (25 mM), 1 μl dNTPs (10 mM), 1 μl Taq DNA polymerase (5 U/μl), 5 μl 10×PCR buffer (100 mM Tris‐HCl, pH 8.8, 25℃, 500 mM KCl, 0.8% (v/v) NP‐40), and 37 μl ddwater. PCR was conducted using the following thermocycling protocol: 94℃ for 5 min, then the optimal number of cycles of 94℃ for 30 s, 56℃ for 30 s, and 72℃ for 1 min. A final extension was performed at 72℃ for 2 min. In addition, all PCR products were further concentrated by ethanol precipitation (as described in 1.2).

To determine the optimal number of cycles, PCR products were assessed every two cycles from 9 to 27 cycles. The products were checked by 2% agarose gel electrophoresis stained with Gel‐red, and the optimal number determined from the results observed on a Gel Doc™ EZ (Bio‐Rad, USA). This number of cycles was then used to enrich the DNA.

#### 4.2.2 Oligonucleotide purification and recovery

##### DNA purification using 10% dPAGE Gel

The DNA library and primers were purified separately. DNA sequences were diluted with ddwater to 10 μM. Ten percent denaturing polyacrylamide gel electrophoresis (dPAGE) was used to purify the DNA using 1×TBE at 120 volts (V) for 45 min for the DNA library and 20 min for primers. The gel was observed using UV light. Target bands were cut into pieces and macerated, and 300 μl DNA elution buffer added. EP tubes were placed on a thermo‐mixer and mixed at 500 r/min at 37℃ for 1 hr. The samples were then centrifuged at 13,800 *g* for 5 min at 4℃, after which 200 μl DNA supernatant was aspirated and transferred to fresh 1.5 ml EP tubes.

##### DNA recovery by ethanol precipitation

Sodium acetate (3 M, pH 5.2) was added 20 μl. A 900 μl volume of ice‐cold 100% ethanol was then added and then thoroughly mixed prior to cooling to −20℃ for 1h. After centrifugation at 12,000 r/min for 20 min at 4℃, the supernatant was discarded. The sample was rinsed in 70% ice‐cold ethanol and again centrifuged. Finally, the sample was dried in a vacuum drying centrifuge at 37℃ for 20–30 min, until the ethanol had completely evaporated.

#### 4.2.3 Preparation of CEMs

*Pseudomonas aeruginosa* was cultured in Luria‐Bertani broth (LB) at 30℃ and agitated at 180 r/min until the optical density at 600 nm (OD_600_) was approximately 1.0. The broth was centrifuged at 13,800 *g* for 5 min at room temperature from which the supernatant was collected as a CEM. The CEM was stored at −20℃ until required for additional experimentation. In addition, the number of *P. aeruginosa* cells was counted using a counting plate method. Other bacteria were cultured using optimal growth conditions, and CEMs similarly collected.

#### 4.2.4 In vitro selection procedures

##### Linking magnetic beads with the library

Fifty microliters of streptavidin (50 mg/ml) coated magnetic beads (0.5 μm in diameter, fully shaken, and resuspended prior to use) were mixed with 500 μl Buffer Ⅰ in a centrifuge tube. The magnetic beads were fully resuspended then separated magnetically. This procedure was repeated three times to wash the magnetic beads. The purified PCR product containing the biotin tag was dissolved in 500 μl Buffer I and mixed with magnetic beads (5 mg/ml). The mixture was placed on a DNA mixer and incubated at 37℃ for 30 min. It was then placed in a magnetic separator for 1 min, after which the supernatant was removed.

##### Positive screening

The magnetic beads were suspended in 150 μl of Buffer Ⅱ. A 150 μl aliquot of the CEM of *P. aeruginosa* (CEM‐PA) was then added. Screening was performed on a DNA mixer at 37℃ for 1 hr. In rounds 1–5 of screening, the duration of reaction was 1 hr. In subsequent rounds, the reaction time was 50, 40, and 30 min for rounds 6–8, respectively.

##### Negative screening

Negative screening was performed during the third and fifth rounds. Magnetic beads were suspended in 150 μl of Buffer II. A 150 μl aliquot of CEM was mixed with *V. anguillarum*, *E. coli*, and *S. aureus* and reacted in a DNA mixer at 37℃ for 1 hr. The magnetic beads were separated and washed twice with Buffer II.

##### Creation of each new library and ratio of cleavage

After screening, the tube was placed on a magnetic separator for 1 min and the supernatant collected and alcohol extracted. The precipitate was used for PCR to restore the new library for the subsequent round of screening. A Q5000 micro‐spectrophotometer (Quawell, USA) was used to measure the concentration of the cleavage fragments (concentration = C). All immobilized biotinylated DNA strands (concentration = C_0_) were released from the magnetic beads by the addition of 10 mM EDTA, pH 8.2 in 95% formamide solution, and incubation at 95℃ for 2 min to break the streptavidin‐biotin bonds (Ignjatovic et al., 2010). From this, the cleavage fraction was calculated (C/C_0_).

##### High‐throughput sequencing

The screening process was evaluated by comparing the ratio of cleavage for each round of screening. PCR products were assessed by 2% agarose gel electrophoresis and purified by alcohol precipitation, then dissolved in ddwater. A DNA concentration of >1 μg/μl was used. Sequencing was performed by Sangon Biotech (Shanghai, China).

#### 4.2.5 PAE‐1 DNAzyme sensor based on fluorescence

The DNAzyme sensor molecular beacon was prepared as follows (Ali et al., 2011; Gu et al., 2019). The DNAzyme (10 μM) and FAM‐labeled substrate (15 μM) were heated to 90℃ for 5 min then gradually cooled to room temperature (20℃) to form a DNAzyme complex (5 μM). An 86 μl aliquot of Buffer II, 4 μl of 5 μM DNAzyme complex, and 10 μl of CEM‐PA were mixed in the wells of a 96‐well plate for measurement of kinetics. The fluorescence intensity of each sample was measured in triplicate in a multi‐function full‐wavelength microplate reader (Infinite M1000 Pro, Tecan, Switzerland). Emission at a wavelength of 535 nm was measured at intervals of 30 s for 120 min at an excitation wavelength of 485 nm.

#### 4.2.6 Activity assays by dPAGE

The activity of each candidate DNAzyme was analyzed by dPAGE. Four microliters of 5 μM DNAzyme complex were mixed with 10 μl of CEM and 36 μl of Buffer II then incubated for 30 min at room temperature. Termination buffer (200 μl 6×dPAGE gel loading dye solution and 400 μl 8 M urea) was then added. The samples were separated by 15% dPAGE at 150 V for 1 hr, and the percentage cleavage calculated (cleaved plus uncleaved DNA = 100%) using a Bio‐Rad Gel Doc™ EZ imaging system.

#### 4.2.7 Activity assays with metal ions and pH

The majority of DNA enzymes require metal ions for activity, and different concentrations of metal ions can significantly affect DNAzyme activity (Du, Li, Chai, et al., 2020; Hwang et al., 2016; Zhou et al., 2021). The addition of EDTA (300 mM) to chelate polyvalent metal ions inhibited cleavage. The effects of Na^+^ at different concentrations (0, 30, 60, 90, 120, 150, 180, 210, 240, 300, 400, and 600 mM) on the activity of PAE‐1 DNAzyme were examined. Optimal Na^+^ concentration (30 mM) was used to determine the effect of different concentrations of Mg^2+^ (0, 30, 60, 90, 120, 150, 180, 210, 240, 300, 400, or 600 mM) on the activity of PAE‐1 DNAzyme. Furthermore, the lytic activity of DNAzyme was measured at a gradually increasing pH.

#### 4.2.8 DNAzyme sensor

The PA‐1 DNAzyme was combined with a polystyrene board to fabricate a DNAzyme sensor indicator. Twenty microliters of 5 μM DNAzyme complex were mixed with 35.0 μl Buffer II in a light‐proof tube. A 20 μl aliquot of the mixture was then pipetted onto the polystyrene board which was then dried at 50℃ for 2 hr. To test its effectiveness, 30 μl of CEM‐PA was added to the sensor, while, similarly, 30 μl of LB broth was added to a control sample. Fluorescence was measured when irradiated with a blue‐light transilluminator (Sangon Biotech, Shanghai) or using a microplate reader (excitation 485 nm and emission 535 nm). The sensor was stored at room temperature to assess shelf life.

#### 4.2.9 Detection of *P. aeruginosa* in food

*Pseudomonas aeruginosa* was added to pekoe tea, peach juice, hawthorn juice, and tap water. A control sample without bacteria was measured. A 20 μl aliquot of each sample was added to the sensor and incubated for 10 min. Fluorescence was measured when placed in blue‐light transilluminator or using a microplate reader. Black tea, which has low background fluorescence was spiked with *P. aeruginosa* (6.9 × 10^8^ cfu/ml) in a dilution gradient. Primers for the 126 bp eta gene in *P. aeruginosa* were synthesized (primer 1: GACAACGCCCTCAGCATCA; primer 2: CAGCCAGTTCAGCGACCAA). *P. aeruginosa* DNA with the lowest dilution factor was extracted for PCR (reaction program: 94℃ for 5 min; 94℃ for 30 s; 54℃ for 30 s; 72℃ for 30 s; total of 35 cycles; 72℃ for 2 min final extension) to compare the results with DNAzyme sensor.

## CONFLICT OF INTEREST

The authors confirm that there is no conflict of interests regarding this paper.

## Data Availability

The data that support the findings of this study are available from the corresponding author upon reasonable request.
